# Child protection: a universal concern and a permanent challenge in the field of child and adolescent mental health

**DOI:** 10.1186/s13034-016-0106-7

**Published:** 2016-06-14

**Authors:** Joerg M. Fegert, Manuela Stötzel

**Affiliations:** Child and Adolescent Psychiatry/Psychotherapy, University Hospital Ulm, Ulm, Germany; Independent Commissioner for Child Sex Abuse Issues, Berlin, Germany

For much of history, cruelty to children was viewed as a private rather than a societal concern. An early sign of change occurred in the 1870s, when the pivotal case of Mary Ellen Wilson, a severely abused child in New York City, attracted intensive coverage in influential newspapers such as the New York Times and led to the founding of the first child protection agency. Sociologist Michael King has described the media response to cases of extreme child abuse in terms of a moral agenda, saying, “In this category of agenda it is not individuals, but social systems which are being unjust to children” [1].

It would take many more years before child protection would come to be seen as the responsibility of society overall. In 1889, the British Parliament issued the first law to protect children from abuse; however, for a long time, child protection services remained the domain of private philanthropic societies rather than of the state. Awareness of the problem increased following the publication in 1962 of a revolutionary article by Kempe et al. titled “The battered child syndrome”[2], which described clinical evidence of child abuse and emphasized the importance of medical diagnostics in the field of child protection. The effect of this publication was to launch an organized movement within the medical profession to intervene in cases of child abuse and neglect.

More recently, fatal cases of abuse such as those of eight-year-old Victoria Climbié in the UK in 2000 and a two-year-old boy identified only as “Kevin” in Germany in 2006 motivated many countries to launch serious investigations into dysfunctions in the child welfare system, and inspired new legislation and changes in child protection practices. Revelations about the sexual abuse scandals of the Roman Catholic Church and various educational institutions further contributed to a change in the public’s perception of child sexual abuse, physical abuse, and neglect. Media coverage of the scandals and the ensuing political reactions helped drive advances in research, and the last 20 years has seen a vast increase in scientific publications in this area.

While individual tragedies have served to attract attention to child maltreatment, focusing on single cases can be a hindrance with respect to acknowledging the magnitude and ubiquity of the problem. What is needed are ongoing efforts, supported by adequate funding, to conduct fundamental research into the prevention and the consequences of traumatization in childhood. These efforts need to include the implementation of monitoring systems and epidemiology studies, so that data can be monitored and assessed in a comparable way across different countries. Prevention and intervention strategies need to be developed, and approaches that are found to be successful need to be implemented on a larger scale. Some efforts that have been made in recent years to address the problem at the international level and within Germany are described below.

Back in 2000, the United Nations set out its “Millennium Development Goals”, which over the following years helped to contribute to a worldwide reduction of child mortality, better maternal health, and other medical improvements. Building on this example, in 2015 the U.N. released a report titled “The Road to Dignity by 2030: Ending Poverty, Transforming All Lives and Protecting the Planet”, which laid out an agenda for formulating sustainable development goals through the promotion of peaceful and inclusive societies. One of the important goals in this agenda is “to end abuse, exploitation, trafficking and all forms of violence and torture against children” [3]. This report was followed by one put out by UNICEF titled “A Post-2015 World Fit for Children” [4]. The first agenda item in the UNICEF report reads as follows:

*“****End violence against children:****In a world where almost one billion children under 15 suffer regular physical punishment, and nearly a quarter of all girls between the ages of 15 and 19 report experiencing physical violence, violence against children affects every country and every community. While violence against children is often invisible, its impact on individual children and their societies is profound and far*-*reaching, undermining developmental gains made in other areas. Because violence against children is a universal problem, investing in protecting children from violence, exploitation and abuse must be a global priority. More must be done to raise awareness of violence and encourage people to speak out when they see or suspect violence against children and to strengthen social welfare systems and services that protect children from harm and provide support to those who are already victims of violence.”*

The World Health Organization (WHO) has also taken steps to address violence against children, issuing regional reports on the prevention of child maltreatment. In the report issued for Europe in 2013 [5], it proposed that all European countries develop national policies based on multi-disciplinary efforts, and that they define priorities for research. It also urged improvements in data collection for purposes of monitoring and evaluation, since such knowledge is of utmost importance in strengthening the ability of health systems to implement strategies for prevention and treatment. In addition, the WHO has produced a Toolkit for mapping the responses by legal, healthcare, and social services to child maltreatment [6].

The European Union sponsored a project titled CAN–MDS (*C*oordinated Response to Child *A*buse and *N*eglect via a *M*inimum *D*ata *S*et) aimed at coordinating the monitoring of routine data in child protection systems in Europe. However, comparison of national data sets in Europe remains difficult because many studies use different definitions. In 2014, the WHO released a publication titled “Investing in children: The European child maltreatment prevention action plan 2015–2020” [7]. This report estimated that the global prevalence rates for child maltreatment are 16.3 % for physical neglect and 18.4 % for emotional neglect, from which it concluded: “Applying these figures to the population of children in Europe suggests that 18 million children suffer from sexual abuse, 44 million from physical abuse and 55 million from mental abuse.” It further stated, “Child maltreatment is a cause of social and health inequality within and between countries. There is strong evidence for the development of mental and physical disorders. Therefore capacity building in child and adolescent psychiatry and in mental health services for children and adolescents is crucial.”

These high estimates of prevalence rates, which were derived mainly from self-report studies, came as a shock to many clinicians as well as to the public. Their accuracy is not certain, given that data from different sources may not be comparable. However, given the likely magnitude of the problem, all societies should make the strengthening of child protection systems a national priority. Considering the low investment that has been made in research and development up to now, the WHO identifies this problem as a “best buy”, meaning that a great deal of value and advancement can be gained for a relatively small investment compared to that needed to address other health issues.

The societal costs of the consequences of child maltreatment are very high. In Germany, a study sponsored by the Ministry of Family Affairs on the annual costs to society arising from all forms of traumatization in childhood (i.e., neglect, physical abuse, and sexual abuse) found that the estimation based on a moderate model was 11 billion euros [8–10]. Similar costs have been found elsewhere. A US study done in 2008 estimated the societal costs related to child abuse and neglect to be USD 103.8 billion per year, not including intangible costs [9]. In Australia, a 2007 study calculated these costs at approximately AUD 4.0 billion on the basis of a population survey, and at AUD 10.7 billion on the basis of prevalence information from literature [10]. In Canada, a study done in 2003 calculated the “minimum cost to society” to be around CAD 15.7 billion [11]. As these findings were published several years ago, it seems likely that costs have gone up since then.

In Germany, following the child sexual abuse scandal that came to light in 2010, the government appointed an Independent Commissioner to gather data on the problem and provide recommendations [12–14]. In December 2014, in collaboration with the editor of CAPMH and supported by the Dreiländer Institute, a center for research and teaching that serves the three German-speaking countries, the Independent Commissioner invited leading international experts in the field of child abuse and neglect to a meeting in Berlin to discuss ways to implement better monitoring systems [15, 16].

The first article in this series, by Jud et al., is based on a report edited by the Independent Commissioner [17, 18]. That report provides an overview of international research as well as recommendations for the future development of research and monitoring in the field of child protection in Germany. The article by Trocmé et al. analyzes trends in the rate of child maltreatment and of foster care placements in Canada, and describes how there has been an increase in both the number of investigations of child abuse and in the number of children removed from their homes. Glaesmer et al. report on prevalence rates Germany that were derived using the Childhood Trauma Questionnaire [19].

While there is increased knowledge today about the prevalence of abuse, sexual abuse, and neglect of children in different settings, more research is needed on prevention and intervention. Professionals in the fields of child and adolescent psychiatry as well as in other mental health areas can play an important role in establishing a continuous monitoring system within the healthcare system, in cooperation with other professions. The scaling-up of successfully evaluated approaches is one of the greatest challenges. In addition, basic research into genetic and epigenetic effects related to early neglect and traumatization is needed. For example, while the hormonal stress reaction is quite well studied, little is understood yet about the immunological consequences of trauma.

Since 2010, the German government has invested about 50 million euros to assist abused and neglected children, with about 20 million euros going to efforts supporting research and the dissemination of research findings, such as the establishment of e-learning programs aimed at professionals. In 2013, the Editor-in-Chief of CAPMH founded a competence center in the state of Baden-Württemberg for research into child abuse and neglect; and an interdisciplinary trauma research center has been established at the University of Ulm. However, at present there is no agenda at either the national or the EU level for improved coordination of research work and clinical approaches in child protection.

When a working group created by the Independent Commissioner sent out a questionnaire on funding activities and programs to research funding bodies in 2015, the EU was among those that did not respond. It may be that a political agenda such as the “Road to Dignity” report produced by the United Nations is needed in order to underscore the importance of child protection to professionals in healthcare fields. Knowledge about adverse childhood experiences has grown [20], and much is now understood about the lifelong consequences of abuse. Experts in child and adolescent psychiatry and other areas of child mental health are the frontline workers in the diagnosis and treatment of early traumatization.

In recognition of the significance of child abuse, both standard sets of diagnostic criteria have added information specific to this problem. The recently updated version of the Diagnostic and Statistical Manual of Mental Disorders, DSM-5, attempts to integrate more age-specific diagnostic criteria in the definition of PTSD, while the upcoming version of the International Classification of Diseases, ICD-11, due out in 2018, will outline the importance of complex and sequential traumatization to which children who are institutionalized or in foster care are sometimes subjected.

The editorial board of CAPMH invites researchers and clinicians from around the world to publish their findings on child protection in our open access journal. Child protection is an interdisciplinary issue, and open access publishing is the most appropriate way to ensure that data and information are made accessible to everybody.
